# Instability in Evolutionary Games

**DOI:** 10.1371/journal.pone.0049663

**Published:** 2012-11-29

**Authors:** Zimo Yang, Tao Zhou, Pak Ming Hui, Jian-Hong Ke

**Affiliations:** 1 Web Sciences Center, University of Electronic Science and Technology of China, Chengdu, People's Republic of China; 2 Department of Physics, The Chinese University of Hong Kong, Shatin, Hong Kong, People's Republic of China; 3 College of Physics and Electronic Information Engineering, Wenzhou University, Wenzhou, People's Republic of China; Hungarian Academy of Sciences, Hungary

## Abstract

**Background:**

Phenomena of instability are widely observed in many dissimilar systems, with punctuated equilibrium in biological evolution and economic crises being noticeable examples. Recent studies suggested that such instabilities, quantified by the abrupt changes of the composition of individuals, could result within the framework of a collection of individuals interacting through the prisoner's dilemma and incorporating three mechanisms: (i) imitation and mutation, (ii) preferred selection on successful individuals, and (iii) networking effects.

**Methodology/Principal Findings:**

We study the importance of each mechanism using simplified models. The models are studied numerically and analytically via rate equations and mean-field approximation. It is shown that imitation and mutation alone can lead to the instability on the number of cooperators, and preferred selection modifies the instability in an asymmetric way. The co-evolution of network topology and game dynamics is not necessary to the occurrence of instability and the network topology is found to have almost no impact on instability if new links are added in a global manner. The results are valid in both the contexts of the snowdrift game and prisoner's dilemma.

**Conclusions/Significance:**

The imitation and mutation mechanism, which gives a heterogeneous rate of change in the system's composition, is the dominating reason of the instability on the number of cooperators. The effects of payoffs and network topology are relatively insignificant. Our work refines the understanding on the driving forces of system instability.

## Introduction

Instabilities are widely observed in diversified fields such as sociology, psychology, economics, and biology [1]–[19]. For example, biological evolutions exhibit themselves as intermittent bursts of activities separating relatively long periods of quiescence, with extinctions happening at all scales [5], [6]. This dynamical instability, referred to as punctuated equilibrium, may result from strong interactions among different species [7], [8]. Economic crises, an instability phenomenon in economic systems, are caused not only by the economic and financial policies of individual country, but also the interdependent relations among countries, known as the world trade network and other economic and financial networks [14]–[17].

These systems typically consist of many interacting individuals, each reacting to the environment and other individuals' actions to enhance its own benefit. The relationship among individuals can be described by a network, with nodes and links representing the individuals and their relations, respectively [20], [21]. The reacting strategies are usually modeled by competing games, which were introduced for biological problems [22], [23] and subsequently applied to many other disciplines [24]–[28]. Therefore, a combination of evolutionary games and networks provides an effective approach of research on these systems [29]. To get closer to reality, the mechanisms of “network evolution” [30], [31] and “inheritance and variation” [32], which can also be called imitation or copying mechanisms, were incorporated into subsequent research.

Understanding the underlying mechanisms for system instability has been the focus of recent research. Kim *et al.* [33] pointed out that an opinion leader could affect a considerable fraction of population yet ordinary people can rarely influence the leader, and this kind of asymmetric influence could result in dynamic instability in prisoners' dilemma game. Schweitzer *et al.* [15] showed that a single tiny disturbance may lead to the system-level instability through the cascading process on economic networks. Rendell *et al.* argued that the copying and learning mechanisms would result in instability [34]. Cavaliere *et al.* [35] proposed a game-theoretic model of dynamic network formation for studying prosperity and instability in which newcomers are more likely to select prosperous individuals as role-models and imitate their strategies and connections. Their model incorporates three mechanisms: (i) imitation and mutation, (ii) preferred selection on successful individuals, and (iii) networking effects, and can exhibit instabilities on both the composition of individuals and the interacting patterns of individuals.

While these mechanisms combined could lead to the system instability, the effects of each individual mechanism are not fully understood. In particular, is there a dominating mechanism for the instability on the composition of individuals? Here, we propose and study simplified models to distinguish the contributions of each mechanism. It is found that imitation and mutation alone can lead to the instability on the composition of individuals in a symmetric way, and the preferred selection mechanism modifies the instability and makes the system exhibit asymmetry. Surprisingly, the co-evolution of network topology and game dynamics is not necessary to the occurrence of instability, in particular, if the new links are added in a global manner, the network topology exerts almost no impact on such instability. The results are further supported by analyzes based on mean-field approximation. This work, therefore, enhances our understanding on the driving forces of system instability.

## Results

Our models are constructed under the framework of the *snowdrift game*, yet qualitatively the same phenomena result also in corresponding models using the *prisoner's dilemma* (see the **Supporting Information** for results on prisoner's dilemma). The snowdrift game [36]–[39] is best illustrated by a situation where two drivers are caught in a blizzard and blocked by a snowdrift. Each driver has two choices: either removing the snowdrift by shoveling or staying in the car. If the road is cleared, both drivers get a benefit 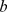 of getting home. There is a cost 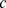 for the labor of shoveling, with 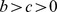. If the drivers cooperate in clearing the block, they share the labor and each gets a net benefit of 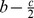. If both choose to stay in the car, they both get zero benefit. If one of the drivers shovels, then both can go home, but the non-cooperative driver (defector) avoids the labor and gains a benefit 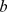, whereas the cooperator's benefit is 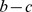. Writing 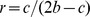, the model can be described by the payoff matrix [40]:
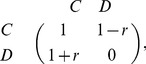
(1)where 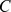 and 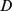 denote the strategies, say cooperate or defect, of the drivers. In a networked environment, the payoff to an individual in a time step is the sum of payoffs from pair-wise interactions with all her neighbors.

### Imitation and Mutation

Considering a system of 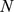 individuals, each of which takes on one of two strategies: cooperate or defect. In every time step, a new individual enters the system, chooses a role-model *randomly*, and imitates the role-model's strategy with a probability 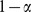 or adopts the opposite strategy with probability 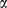. The parameter 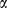 is thus called the mutation rate and 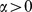 is needed to avoid the system from being frozen into a state with all the individuals using the same strategy. To keep 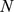 constant, a randomly chosen individual is removed from the system at the same time. This process is very similar to the well-known *Moran process* [41] and thus can be considered as a variant of the Moran process (the Moran process does not take into account the mutation rate, corresponding to the case of 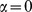). In the supplementary information of [35], Cavaliere *et al.* provided detailed analysis about the differences between birth-death updating and death-birth updating rules. Comparing with the model of Cavaliere *et al.* [35], we isolated the effects of imitation and mutation, as the details of the game and thus the payoff and performance of individuals as well as networking effects are all irrelevant. As the statistics are independent of initial configurations, we set the initial condition to be 50% cooperators and 50% defectors.

**Figure 1:**
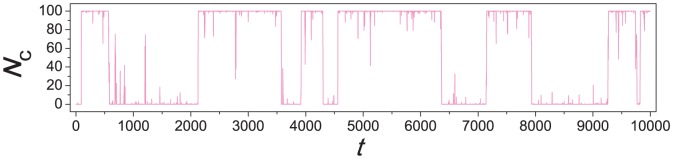
Transitions between extreme states consisting of all cooperators and all defectors. The system size is 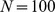 and the mutation rate is 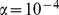. The simulation was carried out for 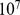 time steps. Each data point is an average over 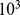 time steps.

**Figure 2:**
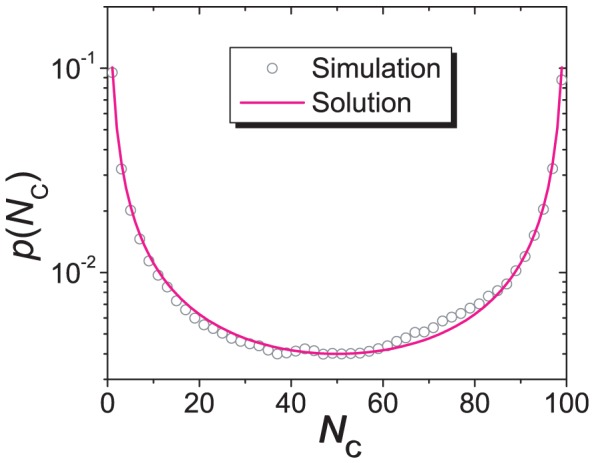
Simulation and analytic results of the distribution 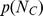 of the number of cooperators. Results are obtained in a simulation of 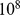 time steps (violet open circles). Considering time steps with 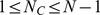, the value of 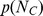 are found as the fraction of steps with exactly 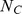 cooperators. The parameters are the same as those in Figure 11. The red solid line represents the the analytic results as given by Eq.(2).

**Figure 3:**
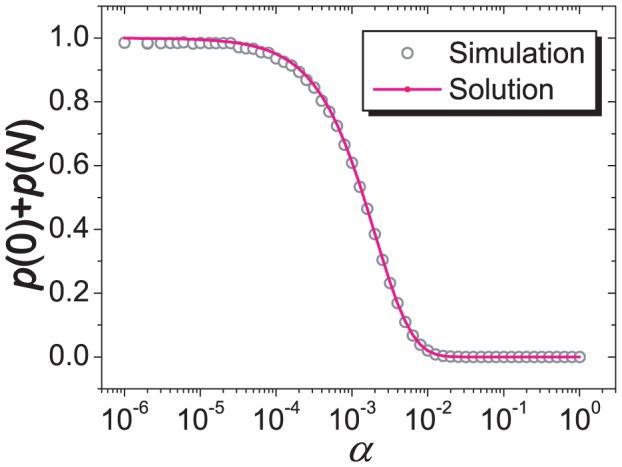
Relative abundance of the system being in states of all cooperators and all defectors as a function of the mutation rate 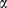. The system size is 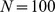 and each data point is obtained by a simulation of 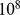 time steps. The purple dash line represents the analytic solution given in Eq.(10).

Figure 1 shows how the number 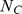 of cooperators varies in time. It is observed that for a majority of time steps, most individuals in the system take on the same strategy, but instability sets in to swing the system to the opposite strategy. A state between the two extremes does not stay long. Excluding the time in uniform states where all individuals take on the same strategy, figure 2 gives the probability density function 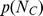 of having 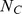 cooperators in the system. The distribution is symmetrical around 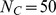, and the system spends much more time when there are many cooperators or defectors than when there are comparable numbers of them. Analytically, a rate equation approach (see **Analysis** for details) gives
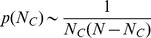
(2)for 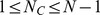, and it gives good agreement with simulation results. This result does not depend on the mutation rate 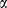 as long as 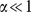. The value of 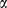 does determine the relative abundance of the two extreme uniform states, with a smaller value giving a larger 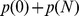, as depicted by the simulation and analytic results in figure 3 (see **Analysis** for analytic treatment). The results in figure 1 to figure 3 show that the imitation and mutation mechanism alone would lead to instabilities on the composition of individuals (quantified by the number of cooperators 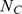). As the selection is made randomly, there is no preference on cooperators or defectors, resulting in a symmetric 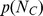 around 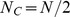.

### Selection Mechanism

To study the effects of preferential selections, we incorporate the snowdrift game into the model. At every time step, each individual plays the snowdrift game with all her connected neighbors and gets a total payoff according to the payoff matrix (1). Individuals will get different payoffs depending on their strategies and their competing neighborhoods. A newcomer then enters the system and selects an individual 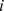 as the role-model with a probability proportional to the total payoff of individual 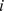. To allow individuals with vanishing payoffs to have a chance to be chosen, we add a small amount 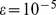 to every individual's total payoff (see **Analysis** and **Supporting Information** about the effects of 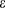 on analytical treatment and numerical results, basically speaking, it has almost no impact if 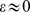). The newcomer will follow the role-model's strategy with probability 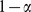 or adopts the opposite strategy with probability 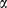, where 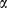 is the mutation rate. After deciding on the strategy, the newcomer establishes 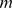 links randomly with existing individuals. The time step ends with the removal of one individual randomly from the 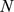 old individuals. Here, we focus on a typical case of 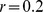 (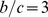), which favors cooperation. Other values of 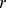 will lead to similar results if 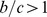.

**Figure 4:**
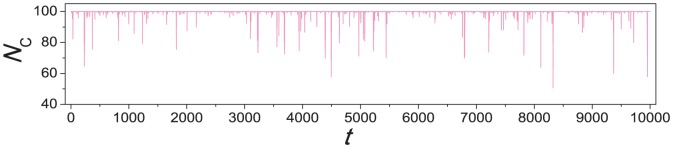
Number of cooperators as a function of time with preferential selection. The parameters are 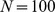, 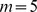, 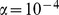 and 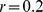. The simulation was carried out for 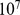 time steps. Each data point is an average over 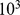 time steps. The system spends most of the time in a state of all cooperators, interrupted by instabilities that last for a short duration when defectors suddenly appear. This is analogous to the coexistence of prosperity and instability as observed in the model of Cavaliere *et al.* [35].

Figure 4 shows the simulation results of 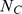 as a function of time. The preferred selection of more successful individuals leads to a dominance of cooperators. However, the system does not stay in a state full of cooperators all the time. There are instabilities resulting in the sudden occurrence of many defectors that last only for a short duration. As a result, the total payoff of all individuals over time is still high. The situation is similar to the coexistence of prosperity and instability in the model of Cavaliere *et al.* [35] (later we will show quantitatively the changes of system profile and strength of instability versus 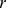, which provide nice evidence on their similarity). Despite the similarity, we stress that the coexistence does not rely on network evolution in the present model, as the 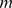 links are established randomly and the network grows independently of the game dynamics, which is different from that in Ref. [35].

### Network Evolution Mechanism

**Figure 5:**
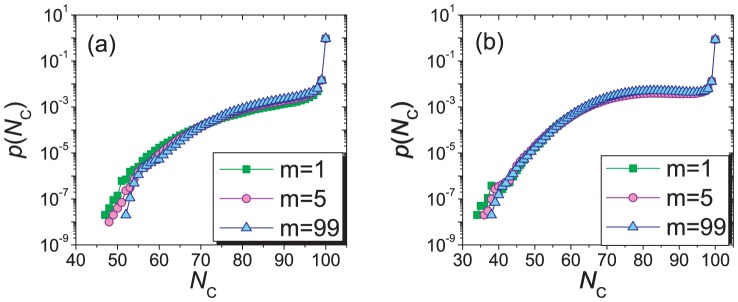
The distributions 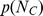 for different 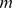. With 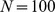 and 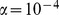, subgraphs (a) and (b) respectively show the time distributions for 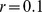 and 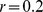. The simulations last for 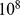 time steps. Distributions for different 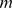 overlap each other, implying that the number of links in the network has no influence on the prosperity of cooperation and the system instability.

**Figure 6:**
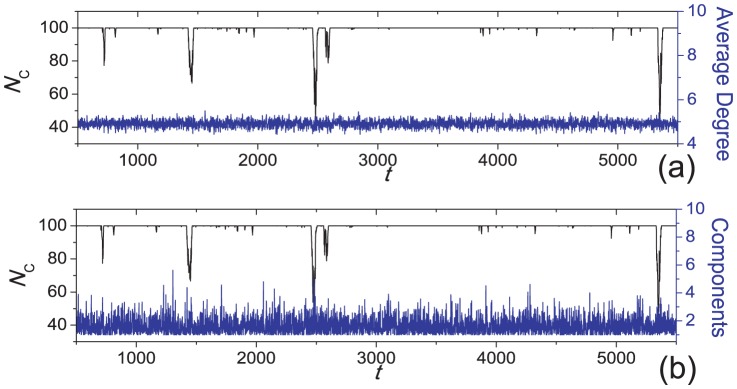
Number of cooperators shown together with (a) the instantaneous average degree of the network and (b) the number of disjoint components in the network. The parameters are 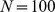, 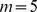, 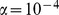 and 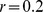. Results are shown for the early stage. Each data point represents an average over 100 time steps.

**Figure 7:**
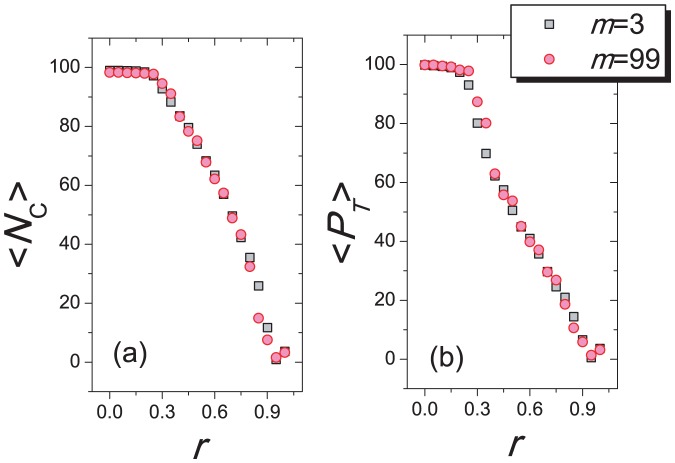
The changes of (a) the average number of cooperators 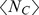 and (b) the average system payoff 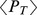 versus parameters 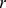 and 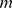. Other parameters are fixed as 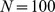 and 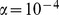. The simulation lasts for 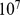 time steps. The black squares and red circles represent the cases of 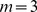 and 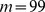, respectively.

The independence on network evolution is further illustrated by considering the distributions 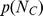 for different values of 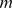. Figure 5 shows that the distributions 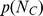 for 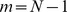 corresponding to a fully connected network, 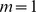 corresponding to a network fragmented into small pieces and 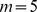 as an intermediate case are almost the same. Analytic result of 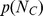 also shows that 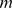 is irrelevant (see **Analysis**). The preferential selection mechanism makes 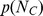 asymmetric and shifts it to the side of larger 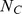, when compared with figure 2. In contrast, the model of Cavaliere *et al.* [35] gives a network that undergoes continual fragmentation and coalescence, which in turn affect the fraction of cooperators in the system. The insensitivity to network topology is further illustrated in figure 6, in which we show time variations of the average degree and the number of disjoint components in the system at short times. These quantities vary in a random fashion, with no observable correlation with 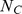. Figure 7 reports how the average number of cooperators 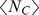 and the average system payoff (i.e., the total payoff of all individuals) 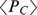 change with parameters 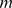 and 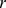. Again, 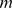 has almost no impact on either 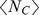 or 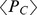 and we display two examples 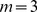 and 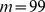 in figure 7.

To further demonstrate that the network evolution mechanism is not a necessary factor leading to the features in figure 5, we study some other mechanisms like *good-get-richer* [42]–[44], where the newcomer has a probability 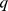 (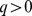) to connect to the role-model, in addition to a higher probability of selecting the individuals with higher payoffs as role-model, and a probability 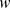 (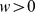) to connect to other individuals. Under such good-get-richer mechanism, the network evolution is related to the game dynamics, but it still gives almost the same distribution 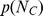. Typical simulation results are presented in Fig. S1. Notice that, in the present model, the links are always added in a global manner, while in the model of Cavaliere *et al.* [35], the links are added in a localized manner. Therefore, one could infer that the different ways of network construction indeed matter, but the constructing rule is not necessary to be one of the origins of instability.

### Effects of Payoff Matrix

In accordance with the mechanism of the snowdrift game, as the increasing of 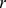, defectors are encouraged and the number of cooperators decreases, leading to the decrease of the system payoff 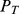. This monotonous changes are illustrated in figure 7.

**Figure 8:**
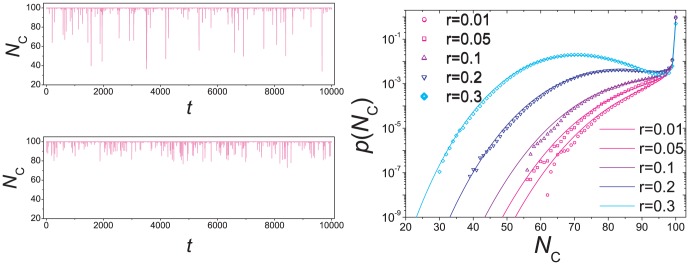
Effects of payoff parameter 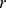 on 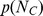. Number of cooperators as a function of time for (a) 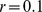 and (b) 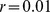. The data are obtained from simulations and the lines come from numerical solutions. The parameters are 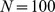, 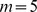 and 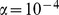. Each data point represents an average over 1000 time steps. (c) Distributions 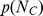 for different values of 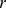. The data points are simulation results and the lines are analytic results.

As shown in figure 8, with preferential selections, insensitive to different values of 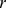, the system is dominated by cooperators in most time with short-duration instabilities. The number of defectors in the instabilities increases with 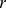, resulting in the quantitatively different 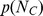 as shown in figure 7(c). Analytically, 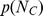 can be obtained using the mean-field approximation. Results are also shown in figure 7(c). The result indicates that (i) 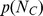 does not depend on the network topology and (ii) the distribution 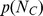 depends on 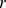, in agreement with simulation results. More detailed simulation results on the effects of 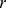 are given in Fig. S2. Analytic treatment is presented in **Analysis**.

**Figure 9:**
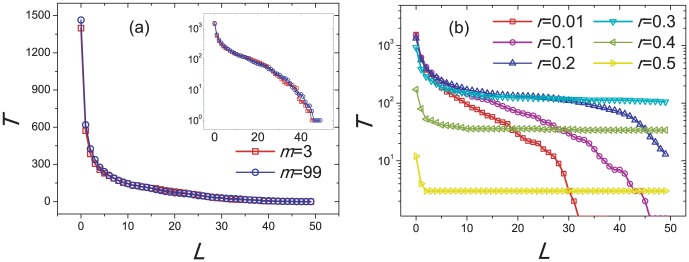
How the strength of instability 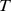 changes with the threshold 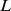 for different 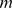 and 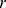. The parameter 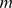 has almost no impact on the strength of instability and the plot (a) compares two examples 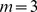 and 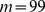, meanwhile 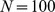, 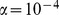 and 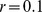 are fixed. Inset of the plot (a) displays the same curves in log-linear scale. The plot (b) shows the considerable effects of 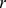 on the strength of instability. In fact, 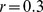 is a borderline: when 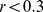 the tails of 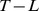 curves will decay quickly for large 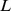, namely the change of the composition of individuals is less drastic, while if 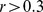, the strength of instability decreases as the increase of 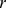, which is of the similar varying tendency to the system payoff. Other parameters are 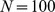, 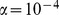 and 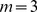. All simulations lasts for 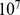 time steps.

For the present dynamical process, we quantify the strength of instability on the composition of individuals by counting the total number 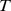 of transitions between all-cooperator state and all-defector state, sharp drops from all-cooperator state and raises from all-defector state (see **Methods** for precise definition). As shown in figure 9, 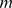 again has almost no impact on the strength of instability, while when 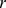 exceeds about 0.3, the strength of instability decreases as the increasing of 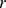 for a wide range of the threshold 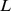. Recalling figure 7, when 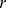 exceeds about 0.3, the average system payoff 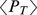 starts to decrease. Though this paper concentrates on the analysis of the instability about the composition of individuals, the observation about how 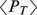 and 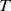 change with 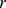 is to some extent similar to the coexistence of prosperity and instability reported in [35].

Furthermore, dynamic instability on the number of cooperators is also observed for the prisoner's dilemma, which is associated with different payoff matrix and different selection mechanism (an individual's payoff can be negative and thus we cannot simply apply the linear selecting probability). See simulation results in figure S3. In accordance with the well-known conclusion, in the well-mixed case (i.e., large 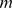), the defectors get dominant.

## Analysis

For the simplest model involving only imitation and mutation, let 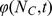 be the probability of having 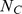 cooperators at the time step 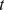. The averaged probability 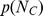 of having 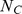 cooperators can be obtained by averaging 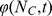 over a sufficiently long time window 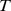 after the transient, i.e.,
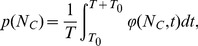
(3)where 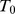 is some time after the transient behavior, which is dependent on the initial condition, ends. A newcomer has a probability 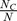 of choosing a cooperator and a probability 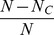 of choosing a defector as the role-model. Therefore, the newcomer has a probability 
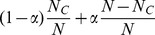
 of taking on the cooperative strategy. In removing an individual, the probability of eliminating a cooperator is 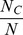, and that for a defector is 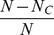. In one time step, the rates 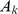 and 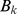 at which a system with exactly 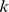 cooperators would evolve into one with 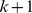 and 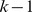 cooperators are given, respectively, by
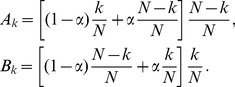
(4)In the steady state, the rates at which 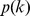 increases are balanced by those at which 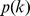 decreases. This results in the following set of equations:
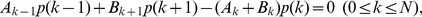
(5)with the first two terms accounting for an increase in 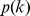 and the last term accounting a decrease in 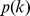. Since 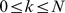, this set of equations can be solved with the supplementary (boundary) conditions 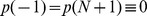. Applying Eq.(5) repeatedly to different values of 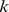, we have in general 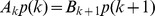. Therefore, Eq.(5) can be solved exactly to yield
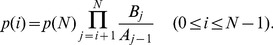
(6)The normalization condition 
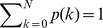
 serves to fix 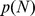 as
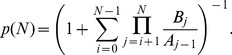
(7)It follows from Eq.(4) 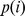 that
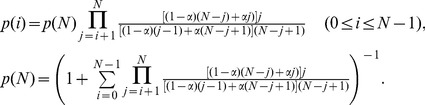
(8)This exact solution exhibits several interesting features. In general, 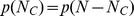 (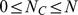). Thus 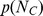 is symmetric around 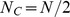, in agreement with that observed in figure 2. For values of 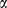 with 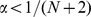, 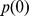 and 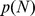 corresponding to the all-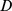 and all-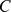 states are more probable. For small values of 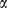, i.e., 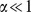, Eq.(8) gives approximately
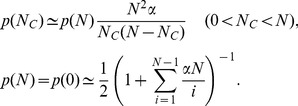
(9)
Equation (9) gives the function form of 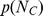 (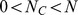) in figure 2. Equations (8) also gives 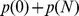 as a function of 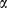, as studied in figure 3. In particular, for 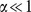, Eq.(9) gives
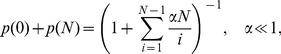
(10)which gives the correct behavior as shown in figure 3. In addition, Eq.(8) indicates that for the particular value of 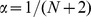, 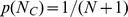 for all 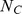. For 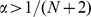, a bump starts to appear around 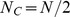 and behaves asymptotically as a gaussian distribution.

Next, we consider the model in which a newcomer selects a role-model preferentially and establishes 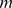 connections randomly. Within a mean-field approximation, we assume that all the cooperators have the same competing environment and all the defectors have the same competing environment. For a cooperator in the system with 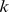 cooperators, there are on average
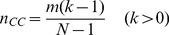
(11)neighbors who are cooperators and 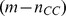 neighbors who are defectors. Similarly, for a defector, there are on average
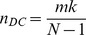
(12)neighbors who are cooperators and 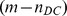 neighbors who are defectors. Therefore, the payoff to a cooperator is 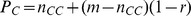 and that to a defector is 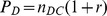. The probability of choosing a cooperator as the role-model is 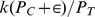 and the probability of choosing a defector is 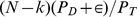, where 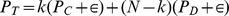 is the total payoff in the system. Here, 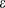 is a small parameter so that every individual would have a finite probability of being chosen as a role-model (as shown in figure S4, the parameter 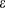 has almost no impact on 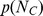 if it is close to zero). Including the effect of the mutation rate 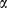 into the case of preferential selection, the rates 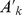 and 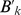 at which a system has exactly 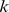 cooperators would evolve into one with 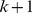 and 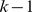 cooperators are given by
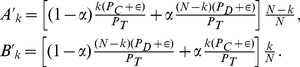
(13)Note that 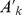 and 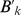 depend on the payoff parameter 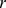. In the steady state, we have in general 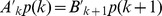, where 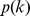 is the averaged probability of having 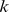 cooperators in the system. Applying the relation recursively to different values of 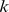, we arrive at
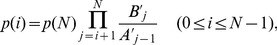
(14)with
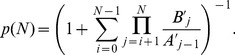
(15)
Equations (14) and (15) have exactly the same form as Eqs.(6) and (7), only with 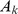 and 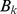 replaced by 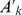 and 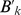. Substituting Eq.(13) into Eqs.(14) and (15) gives 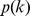 for the case of preferential selection, as that given figure 8(c).

## Discussion

Many complex systems display instabilities during their evolutions, yet the driven force of instabilities may not be as complex as being indicated in the literature. By studying on a series of simplified models, we show that imitation and mutation alone can lead to system instability, while the selection strategy and network structure are relatively insignificant. In particular, the co-evolution of network topology and game dynamics is not necessary for the occurrence of instabilities.

In the extremal situation with most of individuals being in the same state, thanks to the imitation mechanism, the new comer and the removed one are very probably of the same state and the composition hardly changes. Therefore, the system tends to stay long in the extremal situation. Given a game where the cooperators are preponderant in profits and individuals prefer to choose successful ones as their role-models, the imitation mechanism makes the system stay long with cooperators in the dominant position. This is known as prosperity in the literature [35]. At the same time, the mutation rate causes to the instability.

**Figure 10:**
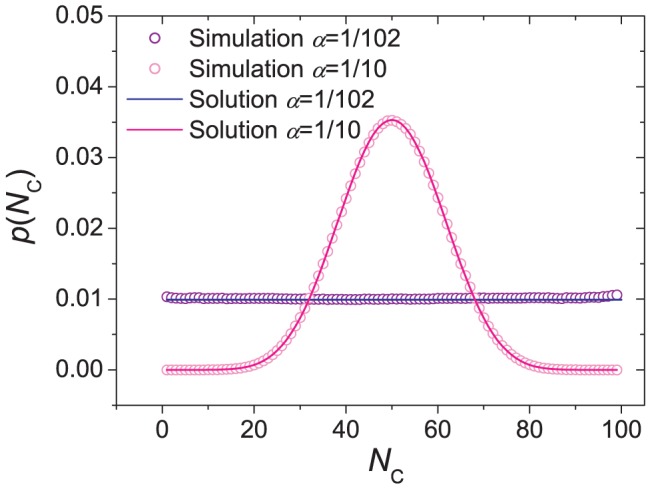
Simulation and analytic results of the distribution 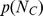 of the number of cooperators for large values of 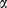. The system size is 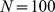 and the mutation rates are 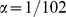 and 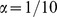, respectively. Simulation lasts for 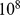 time steps while the analytical solution is presented in Eq. (8).

Notice that, the prosperity and instability coexists only when 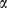 is very small – this is also reasonable otherwise 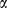 can not be named as *mutation* rate. In fact, when 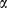 gets larger, the extremal situation will not be preponderant. According to Eq. (8), when 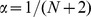, 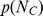 will become fully uniform, say 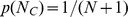 for all 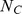. For even larger 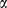, a peak appears at 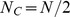 and the distribution behaves like a gaussian function. The results for large 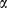 are shown in Figure 10. For an infinite population (i.e., in the thermodynamic limit), 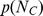 will show a gaussian form for any finite value of 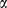. In a word, the instabilities can only be observed for finite-size systems and the critical value 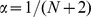 indeed separate two different behavior.

In the present model, no matter the network evolution is independent on (rules presented in the main body) or related to (the good-get-richer mechanism in Fig. S1), links associated with the newcomer are added in a global manner. Therefore, even for far different values of 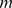, the networks can be all considered as random samplings with different densities from well-mixed population, which is also indicated in the domination of defectors in the prisoner's dilemma game in Fig. S3. In comparison, the new links of the model in [35] are added in a localized way. So one could infer that the way of the addition of links (e.g., globally vs. locally) indeed matters. At least, we arrive to a clear conclusion that the co-evolution of network topology and game dynamics is not a necessary condition to the occurrence of dynamic instability. There are still unsolved issues about the precise understanding of networking effects waiting for further study.

## Methods

**Figure 11:**
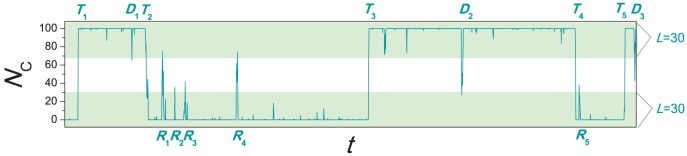
Illustration of how to quantify the strength of instability. We set 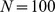 and 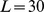. In this specific example, 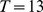, contributed by 5 transitions, 3 drops and 5 raises. Transitions, drops and raises are labelled by 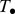, 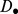 and 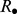 in the plot.

For the present models, we quantify the dynamic instability of the composition of individuals via counting the number of transitions, sharp drops and sharp raises. These three cases are respectively defined as follows: (i) *Transition*.—A transition is a period where the system goes from all-defector state to all-cooperator state but never return to all-defector state during this period or a period where the system goes from all-cooperator state to all-defector state but never return to all-cooperator state during this period. (ii) *Drop*.—A drop is a period where the system goes from all-cooperator state to a state with less than 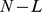 cooperators and then return to the all-cooperator state, during which, it does not reach the all-defector state. Here 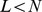 is a threshold. (iii) *Raise*.—A raise is a period where the system goes from all-defector state to a state with more than 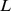 cooperators and then return to the all-defector state, during which, it does not reach the all-cooperator state. Figure 11 illustrates a simple example where 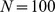 and 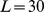. In this example, one could find 5 transition, 3 drops and 5 raises. We use the total number of transitions, drops and raises, 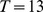, to quantify the strength of dynamic instability. The readers are warned that this definition is suitable for the current case but cannot be directly applied in characterizing instability of a generally dynamical process.

## Supporting Information

Figure S1
**The distributions** 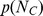 **for different parameters** 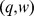 **with good-get-richer mechanism.**
(PDF)

Figure S2
**Effects of** 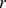 **on the distribution of the number of cooperators** 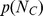 **.**
(PDF)

Figure S3 **Dynamic instability in prisoner's dilemma game.**
(PDF)

Figure S4
**The distributions** 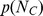 **for different** 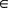 **.**
(PDF)
