# Supplementary material for: Instability in Evolutionary Games
Source: PLoS One. 2012 Nov 29;7(11):e49663. doi: 10.1371/journal.pone.0049663 (PMC3510218; doi:10.1371/journal.pone.0049663)
Supplement: Figure S1 — The distributions for different parameters with good-get-richer mechanism. (PDF) [file pone.0049663.s001.pdf]

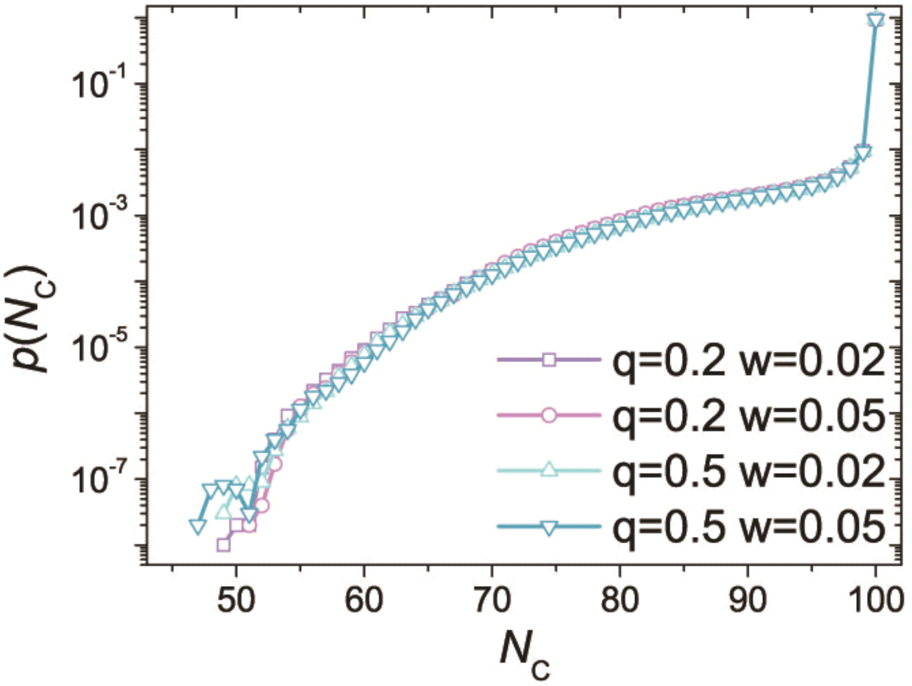

Figure S1. **The distributions $p(N_C)$ for different parameters (q,w) with the good-get-richer mechanism.** Each distribution is obtained by 10^8 time steps with other parameters fixed as N=100, α=0.0001 and r=0.1. The selection strategy tends to select more successful player as the role-model, and when q>w, there is a good-get-richer effect.
