# Supplementary material for: Instability in Evolutionary Games
Source: PLoS One. 2012 Nov 29;7(11):e49663. doi: 10.1371/journal.pone.0049663 (PMC3510218; doi:10.1371/journal.pone.0049663)
Supplement: Figure S2 — Effects of on the distribution of the number of cooperators . (PDF) [file pone.0049663.s002.pdf]

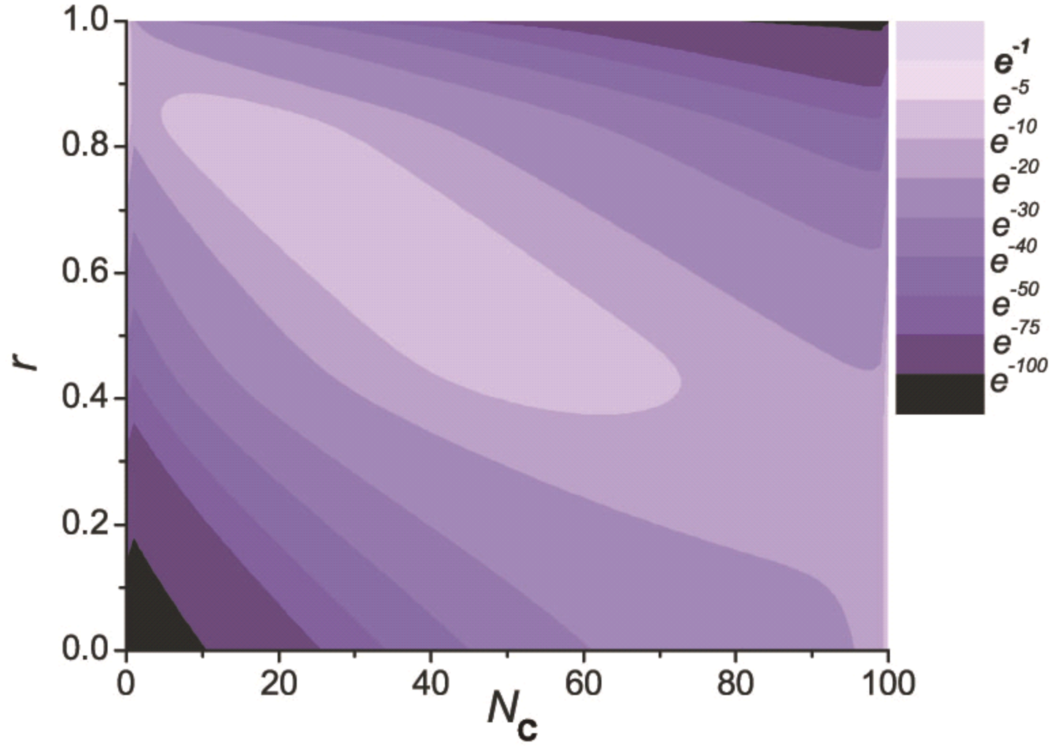

Figure S2. Effects of *r* on the distribution of the number of cooperators $p(N_C)$. This plot shows the analytical solutions from Eqs. (14) and (15) for N=100 and α=0.0001, with the value of $p(N_C)$ indicated by the color. The distribution is normalized as $\sum_{N_C=0}^{N} p(N_C, r) = 1$ for each value of *r*.
