# Supplementary material for: Instability in Evolutionary Games
Source: PLoS One. 2012 Nov 29;7(11):e49663. doi: 10.1371/journal.pone.0049663 (PMC3510218; doi:10.1371/journal.pone.0049663)
Supplement: Figure S3 — Dynamic instability in prisoner's dilemma game. (PDF) [file pone.0049663.s003.pdf]

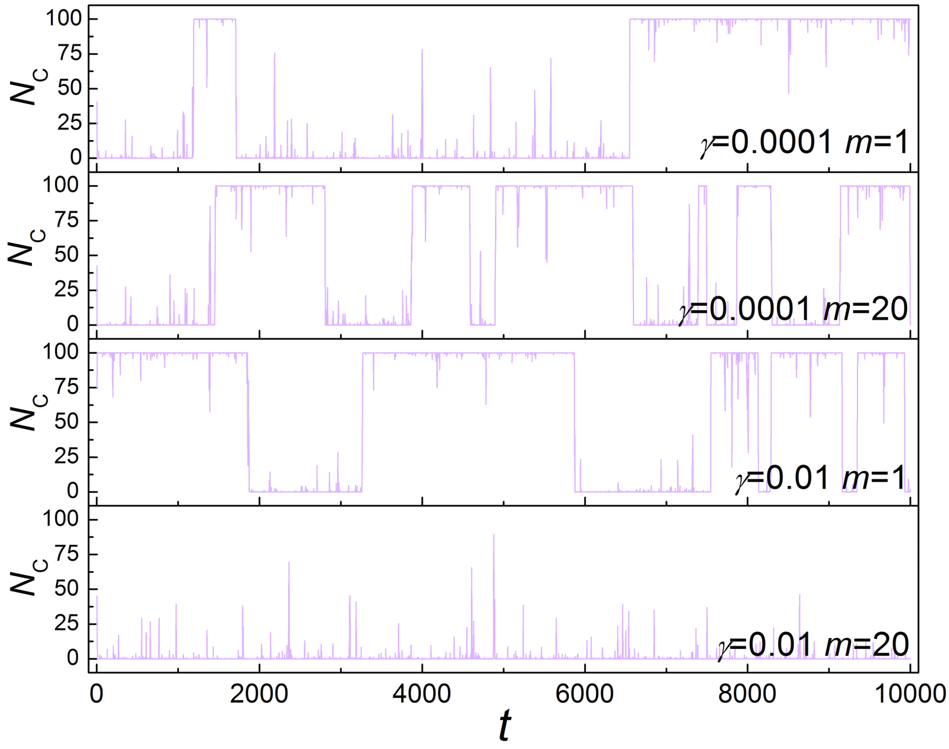

Figure S3. **Dynamic instability in prisoner's dilemma game.** This prisoner's dilemma game corresponds to a payoff matrix (1) with r=1.1. At each time step, a newcomer will choose a role-model and connect to m randomly selected individuals. Other rules are similar to the snowdrift game studied in the main text. Since the payoff can be negative in the prisoner's dilemma game, the selecting probability of an individual is proportional to $(1+\gamma)^{\text{payoff}}$, where γ is a free parameter and the exponent equals her payoff. Other parameters are N=100 and α=0.0001. The simulation was carried out for 10^7 time steps, and each data point is an average over 10^3 time steps. Even with this random connecting mechanism, dynamic instability is still observed. Similar to figure 4, the selection mechanism may lead to asymmetry. In fact, the nonlinear selection mechanism has different effects on the distribution $p(N_C)$ especially in the denser networks. A quantitative and detailed study of these cases is, however, outside the scope of the present article.
