# Supplementary material for: Instability in Evolutionary Games
Source: PLoS One. 2012 Nov 29;7(11):e49663. doi: 10.1371/journal.pone.0049663 (PMC3510218; doi:10.1371/journal.pone.0049663)
Supplement: Figure S4 — The distributions for different . (PDF) [file pone.0049663.s004.pdf]

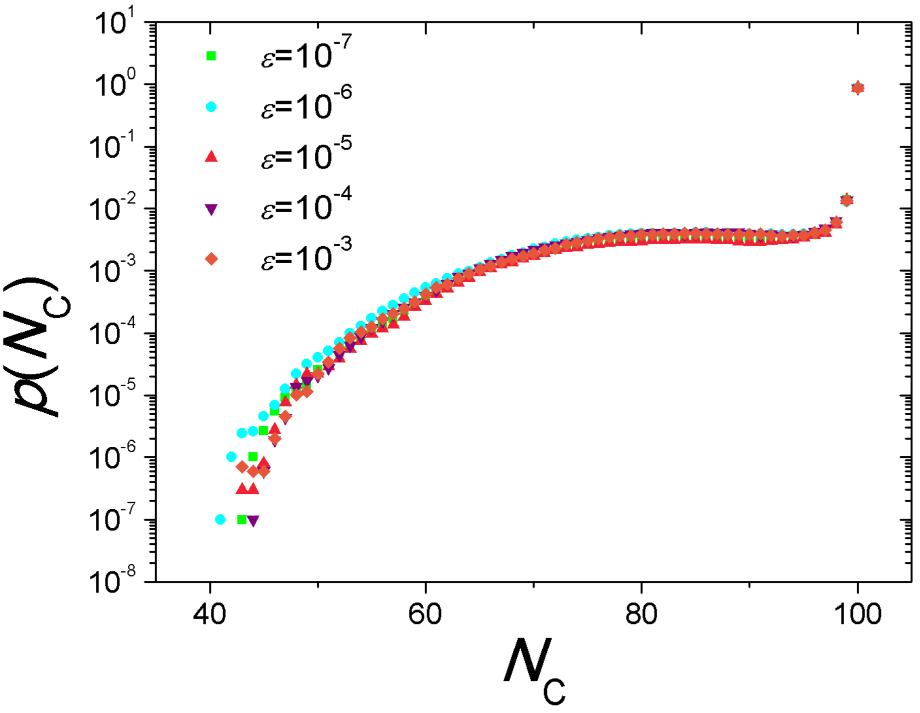

Figure S4. The distributions $p(N_C)$ for different ε. Each distribution is obtained by 10^7 time steps with other parameters fixed as N=100, m=3, α=0.0001 and r=0.2. Obviously, the parameter ε has almost no impact on the distributions $p(N_C)$.
